# VERBAL COMMUNICATION BETWEEN NURSING HOME STAFF AND RESIDENTS WITH DEMENTIA AND APATHY: LANGUAGE ANALYSIS

**DOI:** 10.1093/geroni/igac059.536

**Published:** 2022-12-20

**Authors:** Ying-Ling Jao, Yo-Jen Liao, Diane Berish, Marie Boltz, Wen Liu, Jacqueline Mogle, Helen Kales

**Affiliations:** Pennsylvania State University, University Park, Pennsylvania, United States; Pennsylvania State University, Pennsylvania State University, Pennsylvania, United States; Pennsylvania State University, University Park, Pennsylvania, United States; Penn State, Pennsylvania State University, Pennsylvania, United States; University of Iowa, Iowa City, Iowa, United States; The Pennsylvania State University, University Park, Pennsylvania, United States; University of California, Davis, Davis, California, United States

## Abstract

Communication is fundamental in daily caregiving activities in nursing homes and impacts the quality of care. This study examined the verbal communication of nursing home staff and residents with dementia and apathy during daily caregiving activities. This study used a repeated measures design. Participants included 13 residents with dementia and apathy and 13 staff from two nursing homes. A total of 39 videos were recorded to capture staff-resident interactions during caregiving activities (3 videos for each resident). All video-recorded communication was transcribed and segmented into phrases. Bivariate correlations were used for preliminary analysis. The average length of each interaction was 7.5 minutes (range=1.5-12.5). On average, staff verbalized 46.6 words (range=9.3-121.9) with 11.3 phrases (range=3.7-23.4) per minute. Residents verbalized an average of 18.9 words (range=0-83.8) with 4.8 phrases (range=0-17.1) per minute. Unadjusted correlations demonstrated that residents’ phrases per minute were significantly negatively associated with apathy (r=-0.36, p =0.0239), while being significantly positively associated with age (r=0.42, p=0.0071), cognitive function (r=0.42, p=0.0114), caregivers’ words per minute (r=0.44, p=0.0055), and phrases per minute (r=0.42, p=0.0077). After adjusting for clustering by residents, caregivers’ words and phrases remained significantly positively associated with residents’ phrases. Findings suggest that higher resident apathy and cognitive impairment are associated with fewer verbalizations. When caregivers verbalize more phrases and words, residents are also likely to use more phrases. Findings identify the high-risk population for lack of verbal expression and emphasize the importance of staff verbal communication to promote verbal communication in nursing home residents with dementia and apathy.

